# Case Report: Prune perineum syndrome: a rare case with an unfavourable outcome

**DOI:** 10.12688/f1000research.8246.2

**Published:** 2016-06-10

**Authors:** Roberto I. Lopes, Francisco T. Dénes, Gustavo B. Messi, Marcos G. Machado

**Affiliations:** 1 Division of Urology, University of São Paulo Medical School, São Paulo, Brazil; 2 Division of Urology, The Hospital for Sick Children, Toronto, Canada

**Keywords:** Prune perineum syndrome, pelvic malformations, congenital disease, urinary tract

## Abstract

Prune perineum syndrome (PPS) is a rare anomaly, with only two previous case reports, both dying in the perinatal period. We report the first case of PPS that reached childhood.

The patient presented with a hypoplastic genitalia and bilateral cryptorchidism. There was no evidence of an anal orifice. A significant prune-like mass was observed, extending from the perineum to both gluteal regions and to a cephalic mid-line bony prominence, with a 1cm central orifice that discharged urine. MRI confirmed the previous findings and revealed a right crossed ectopic kidney, intestinal malrotation, a hypoplastic infrarenal inferior vena cava and a hypoplastic right iliac artery. Endoscopic evaluation through the orifice revealed a cavity lined by urothelial mucosa, with a small communication to the anterior urethra in its anterior wall.

A staged reconstruction was planned, with a first-step urinary diversion through a continent abdominal reservoir associated to bilateral orchiopexy. He was discharged from the hospital three weeks later under intermittent catheterization. The next surgical step would be the resection of the perineal mass and its cavity associated to the removal of the prominent sacrococcygeal bones. Unfortunately, four months after the first surgery the patient developed an acute abdomen and was submitted to a laparotomy that revealed a necrotic ileal segment secondary to obstructive adherences. He developed severe malabsorption followed by septic shock, dying five weeks after the procedure.

Due to the lack of literature, there is no consensus for the management of these cases. The wish of the family for a better quality of life and social acceptance, compelled us to perform a urinary diversion, to be followed by a plastic and orthopedic reconstruction. Despite the successful initial result, the patient developed a late abdominal obstruction that was misdiagnosed, precipitating his untimely death five months after the first procedure.

## Introduction

Prune perineum was first described in 1979 by Peeden *et al*. ^1^ and to our knowledge only one further case has been presented ^2^. PPS includes significant abnormalities in the development of the caudal axis, with characteristic deformation of the sacrococcygeal spine associated with a large wrinkled perineal mass and a hypoplastic external genitalia. In both previously described cases, the patient died in the perinatal period ^1, 2^. We present the first case of a male born with this disease who reached 5 years of age.

## Case report

The patient was born at the 39 ^th^ week of the uneventful first gestation of a 28-year-old woman, without any family history of congenital malformation or chronic illness. The maternal medical history was uneventful and pregnancy was normal until the last trimester, when control ultrasonography disclosed a fetal perineal abnormality, therefore a cesarean delivery was performed. A large perineal mass, associated to imperforated anus was readily identified. Urine was observed leaking through an orifice in the perineal mass. A left flank terminal colostomy was performed on the second day of life. Post-operatively, the patient presented with good clinical course, with functioning colostomy and preserved renal function, being discharged after 2 weeks. At four years of age, he came to our service without any previous significant clinical problems, including urinary tract infection. The somatic growth and neuro-psycho-motor development were normal. He had no medication, was wearing diapers and a colostomy bag. Despite a small limp, he was able to walk normally.

**Figure 1.**
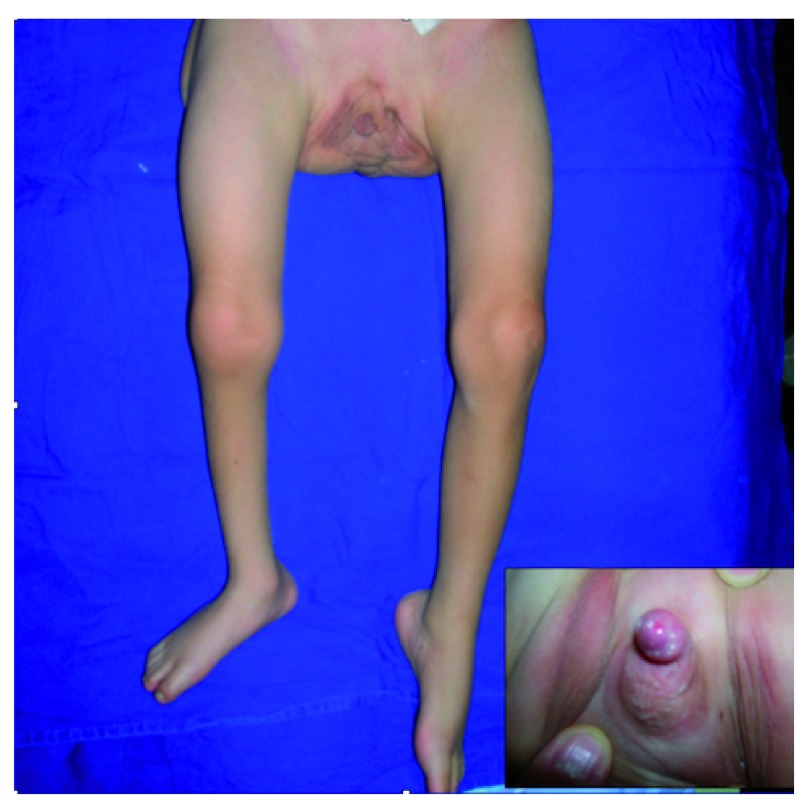
Anterior view of perineum and lower limbs. Hypoplastic external genitals in detail.

**Figure 2.**
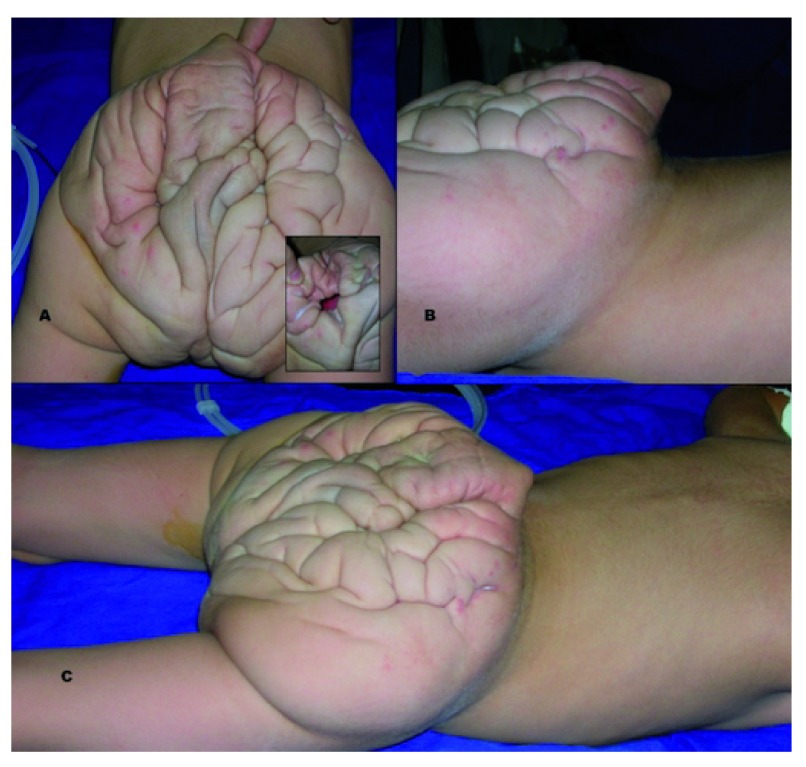
Posterior view showing perineal mass with central orifice in detail ( **A**). Lateral view ( **B**), and perspective ( **C**).

At physical examination, all abnormalities were below the umbilicus. The abdomen had a functioning left colostomy while the genital area presented hypoplastic male external genitalia with bilateral impalpable cryptorchidism. There was no evidence of an anal orifice. The right leg was shorter than the left ( Figure 1). Posteriorly, a 20 × 15 × 8 cm prune-like mass was observed, extending from the perineum to both gluteal regions and to a cephalic mid-line bony prominence, with a 1 cm central orifice that discharged urine. The corrugated skin of the prune-like mass could be easily lifted, disclosing, through its orifice, a large cavity below that contained urine ( Figure 2). In the sitting position, the mass and its cavity were compressed against the perineum.

**Figure 3.**
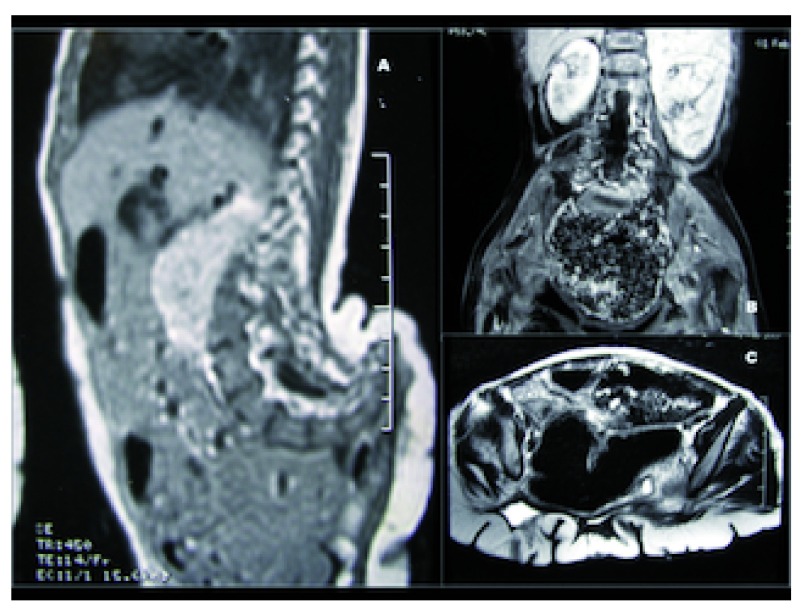
Nuclear magnetic resonance demonstrating exaggerated saccrococcygeal convexity ( **A**); crossing ectopic right kidney and a intestinal malrotation ( **B**); absence of pubic bone, and the corrugated skin covering the posterior perineal cavity ( **C**).

Laboratory examination showed a normal blood count and renal function, and the karyotype was 46, XY. Pelvic radiographs demonstrated an exaggerated sacrococcygeal concavity, lumbar dysrrhaphism and pubic dyastasis. The dorsal bony prominence corresponded to the sacrococcygeal spine, whose abnormal concavity oriented its tip upward, like a small tail. The contrast injection through the orifice of the mass showed a large cavity that extended sagittally and laterally. MRI confirmed the previous findings and revealed a right crossed ectopic kidney, intestinal malrotation, a hypoplastic infrarenal inferior vena cava and a hypoplastic right iliac artery. The posterior concavity of sacrococcygeal bones, associated to a deformity of the pelvic rim was evident, as well as the cavity below the convoluted skin and subcutaneous layer of the perineal mass ( Figure 3).

The endoscopic evaluation through the perineal orifice communicated to a large perineal cavity lined by urothelial mucosa, resembling a bladder that was abnormally located perineally with a small communication to the anterior urethra in its anterior wall. The orifice was at least 1 cm large, and all urine leaked through this orifice. Nevertheless, on cystoscopic evaluation, a guidewire was inserted through the penile urethra and reached the bladder (the urethra was patent, but very thin). As urine was continuously flowing into the perineal cavity (bladder) and leaking through the perineal cavity, it was evident that the ureter (draining alone the right crossed ectopic kidney) reached the perineal cavity. Nevertheless, on “cystoscopic” evaluation we weren't able to find the ureteral orifice.

After extensive evaluation of the case and considering the psychological effects of the malformation, as well as the parental wish for a better quality of life and social acceptance of the child, we planned a staged reconstruction, with a first-step urinary diversion through a continent abdominal reservoir associated to bilateral orchiopexy. We created a continent bowel reservoir, but only one ureter was implanted to it, as the patient had only one kidney. A true bladder was not available and therefore, a continent cutaneous ileal neobladder was performed, with the appendix used for clean intermittent catheterization every four hours in the postoperative care. The presumptive primitive bladder, namely the perineal cavity, was left *in situ*, for posterior removal. It became completely dry after surgery. The procedure was successful and the patient recovered uneventfully. He was discharged from the hospital three weeks later with a dry perineal cavity and under intermittent catheterization regularly performed by the mother. The next surgical step would be the resection of the perineal mass and its cavity associated to the removal of the prominent sacrococcygeal bones. Unfortunately, four months after the first surgery the patient developed an acute abdomen that was misdiagnosed and treated as a urinary tract infection elsewhere. Forty-eight hours later, he was transferred to our service and was submitted to exploratory laparotomy that revealed a necrotic ileal segment secondary to obstructive adherences. The resection of the necrotic segment associated to a terminal ileostomy was performed, but he developed severe malabsorption followed by septic shock, dying five weeks after the procedure.

## Discussion

Previously, only two cases of the rare prune perineum syndrome have been described, and both patients died in the perinatal period due to sepsis ^1, 2^. Early colostomy and normal renal function by an unobstructed solitary kidney enabled long-term survival in our case. Contrary to the two previous cases, this was a male patient with a 46 XY karyotype, although with a very small penis and impalpable testes. An embryologic explanation for the orifice draining urine was searched and we can only postulate that a fistula eventually ruptured between the “perineal bladder” and skin before birth due to increased intravesical pressure (the perineal wrinkled skin was very thin and had no bony support).

Due to the lack of literature, there is no consensus for the management of these unfortunate children. We report the unsuccessful outcome of a child treated for this syndrome. Even though he presented a quite stable physical development without any neurological impairment up to the age of four years, the wish of the family for a better quality of life and social acceptance, compelled us to perform a urinary diversion, to be followed by a plastic and orthopedic reconstruction. Despite the successful initial result, the patient developed a late abdominal obstruction that was misdiagnosed, precipitating his untimely death five months after the first procedure.

## Consent

Written informed consent was obtained from the parent of the patient for publication of this case report and any accompanying images and/or other details that could potentially reveal the patient’s identity.
