# Supplementary material for: Case Report: Prune perineum syndrome: a rare case with an unfavourable outcome
Source: F1000Res. 2016 Jun 10;5:363. Originally published 2016 Mar 16. [Version 2] doi: 10.12688/f1000research.8246.2 (PMC4890296; doi:10.12688/f1000research.8246.2)
Supplement: Peer review history

# Peer review: Referee response for version 2

**DOI:** 10.5256/f1000research.9672.r14291

**Published:** 2016-07-01

**Authors:** Rodrigo L.P. Romao

**Affiliations:** 1 Division of Urology, Division of Pediatric General Surgery, Department of Surgery, IWK Health Centre, Dallhousie University, Halifax, NS, Canada

The added details have improved the report significantly.

 Again, the authors should be congratulated for reporting the best outcome so far for this very severe congenital malformation.

I have read this submission. I believe that I have an appropriate level of expertise to confirm that it is of an acceptable scientific standard.

# Peer review: Referee response for version 2

**DOI:** 10.5256/f1000research.9672.r14293

**Published:** 2016-06-14

**Authors:** Dana Weiss

**Affiliations:** 1 Division of Urology, Departments of Surgery, The Children's Hospital of Philadelphia, Perelman School of Medicine, University of Pennsylvania, Philadelphia, PA, USA

The authors have now provided descriptions of their surgical plan and technique and decision making process. Their report, along with the images they provide, offer a valuable lesson on a way to manage this extremely rare disease.

I have read this submission. I believe that I have an appropriate level of expertise to confirm that it is of an acceptable scientific standard.

# Peer review: Referee response for version 1

**DOI:** 10.5256/f1000research.8868.r14064

**Published:** 2016-05-31

**Authors:** Andrew J. Kirsch

**Affiliations:** 1 Department of Pediatric Urology, Children's Healthcare of Atlanta, Emory University School of Medicine, Atlanta, GA, USA

The authors present a fascinating case of Prune Perineum Syndrome - the first case of survival beyond infancy.  Only two others have been reported with PPS over the past half century. The experienced group of pediatric surgeons describe this exceedingly rare condition and offer the child an opportunity to improve quality of life through surgical reconstruction. The preoperative imaging studies are highly educational and instructive.  Unfortunately, a delayed postoperative complication and poor access to emergency medical care lead to the patient’s demise. This report, however, provides important clinical information to the limited available literature on this condition. This case report will help to educate and guide future generations of patients and their families, as well as physicians and surgeons in the diagnosis and management of PPS.

I have read this submission. I believe that I have an appropriate level of expertise to confirm that it is of an acceptable scientific standard.

# Peer review: Referee response for version 1

**DOI:** 10.5256/f1000research.8868.r12915

**Published:** 2016-05-20

**Authors:** Rodrigo L.P. Romao

**Affiliations:** 1 Division of Urology, Division of Pediatric General Surgery, Department of Surgery, IWK Health Centre, Dallhousie University, Halifax, NS, Canada

The authors report a case of prune-perineum, an extremely rare malformation for which survival beyond the perinatal period has not been documented. Only 2 previous case reports are described in the literature. 

 The description of the findings is interesting and the illustrations are of good quality. The description of the surgical technique is short. If the authors were able to provide more details about the reconstruction performed, it would be of interest to the reader. It is not clear what kind of reservoir was created and how exactly CIC was conducted postoperatively. A detailed account of the findings and intra-operative decision-making process would strengthen the report significantly.

 The authors should be applauded for reporting the best outcome thus far for this exceedingly rare and complex condition.

I have read this submission. I believe that I have an appropriate level of expertise to confirm that it is of an acceptable scientific standard, however I have significant reservations, as outlined above.

# Response

**Published:** 2016-05-31

**Authors:** Roberto Iglesias Lopes

If the authors were able to provide more details about the reconstruction performed, it would be of interest to the reader. It is not clear what kind of reservoir was created and how exactly CIC was conducted postoperatively. A detailed account of the findings and intra-operative decision-making process would strengthen the report significantly.

 Answer: A perineal orifice communicated to a cavity, where we identified a bladder, which was abnormally located perineally. The orifice was at least 1 cm large, and all urine leaked through this orifice. Nevertheless, on cystoscopic evaluation, a guidewire was inserted through the urethra and reached the bladder (the urethra was patent, but very thin). A patent, albeit very thin urethra was connected between the perineal cavity (bladder) and the hypoplastic penile shaft. The bladder neck was also very small.  As the bladder consisted of a cavity lined with urothelium and was not a true bladder, we opted for a continent bowel reservoir. Only one ureter was implanted to it, as the patient had only one kidney. A true bladder was not available and therefore, a continent cutaneous ileal neobladder was performed, with the appendix used for clean intermittent catheterization every four hours in the postoperative care.

# Peer review: Referee response for version 1

**DOI:** 10.5256/f1000research.8868.r13606

**Published:** 2016-05-06

**Authors:** Dana Weiss

**Affiliations:** 1 Division of Urology, Departments of Surgery, The Children's Hospital of Philadelphia, Perelman School of Medicine, University of Pennsylvania, Philadelphia, PA, USA

The authors present a very interesting and unique case report.

Some questions that arise:

Had the child had any renal imaging after birth?  Was the urine leaking through a penile urethra as well as through the orifice that contained urine?  This part is not clear from the description. 

The authors could assist the reader additionally, should this case ever arise again, by describing exactly what kind of reconstruction was done at the first stage. Was a true bladder found? Were the ureters implanted normally into a bladder? Was there a bladder neck and urethra? What exactly was the “orifice” that was draining urine? When the authors say they created a continent abdominal reservoir – was this a bowel reservoir to which the ureters were implanted?  If there was any bladder present, was this used? 

This kind of case report is interesting and important since many pediatric surgeons and urologists may never see something like this in our lifetime, but if we do, it is very valuable to have some inkling of what is going on, and how we might even begin to manage it.

I have read this submission. I believe that I have an appropriate level of expertise to confirm that it is of an acceptable scientific standard, however I have significant reservations, as outlined above.

# Response

**Published:** 2016-05-11

**Authors:** Roberto Iglesias Lopes

**Author Response to Reviewer's Comments:**

Had the child had any renal imaging after birth?

Answer: He was referred to us at age 4, having undergone previously a colostomy elsewhere on the second day of life. We started all diagnostic workup from point zero.

Was the urine leaking through a penile urethra as well as through the orifice that contained urine? 

Answer: The perineal orifice communicated to a cavity, where we identified a bladder, which was abnormally located perineally. The orifice was at least 1 cm large, and all urine leaked through this orifice. Nevertheless, on cystoscopic evaluation, a guidewire was inserted through the urethra and reached the bladder (the urethra was patent, but very thin).

Was a true bladder found?

Answer: Besides the large perineal cavity (identified as bladder) drained by the orifice and connected to the urethra, there was no other structure resembling a normal bladder.

Were the ureters implanted normally into a bladder? 

Answer: As urine was continuously flowing into the perineal cavity (bladder) and leaking through the perineal cavity, it was evident that the ureter (draining alone the right crossed ectopic kidney) reached the perineal cavity. Nevertheless, on "cystoscopic" evaluation we weren't able to find the ureteral orifice.

Was there a bladder neck and urethra? 

Answer: As mentioned before, a patent, albeit very thin urethra was connected between the perineal cavity (bladder) and the hypoplastic penile shaft. The bladder neck was also very small.

What exactly was the “orifice” that was draining urine? 

Answer: We do not have an embryologic explanation for it, and we can only postulate that a fistula eventually ruptured between the "perineal bladder" and skin before birth due to increased intravesical pressure (the perineal wrinkled skin was very thin and had no bony support).

When the authors say they created a continent abdominal reservoir – was this a bowel reservoir to which the ureters were implanted? 

Answer: Exactly: it was a continent bowel reservoir, but only one ureter was implanted to it, as the patient had only one kidney.

If there was any bladder present, was this used? 

Answer: The presumptive primitive bladder, namely the perineal cavity, was left in situ, for posterior removal. It became completely dry after surgery.
